# Evaluating the physiological responses to treadmill exercise based on the 6-minute walk test in individuals with COPD across severity levels

**DOI:** 10.3389/fphys.2025.1668559

**Published:** 2025-10-31

**Authors:** Mohammad Abu Shaphe, Mohammed M. Alshehri, Ramzi Abdu Alajam, Ahmed Ghazwani, Basema Fathi Temehy, Ahmad Sahely, Bsmah Husein J Alfaifi, Ali Hakamy, Ashfaque Khan, Aafreen Aafreen, Abdur Raheem Khan

**Affiliations:** ^1^ Department of Physical Therapy, College of Nursing and Health Sciences, Jazan university, Jazan, Saudi Arabia; ^2^ Department of Respiratory Therapy, College of Nursing and Health Sciences, Jazan University, Jazan, Saudi Arabia; ^3^ Department of Physiotherapy, Integral University, Lucknow, India

**Keywords:** chronic obstructive pulmonary disease, 6-min walk test, treadmill exercise, exercise intensity, disease severity, rehabilitation

## Abstract

**Background:**

Chronic obstructive pulmonary disease (COPD) limits exercise capacity and impacts lifestyle. While treadmill exercise aids rehabilitation, the 6-Minute Walk Test (6 MWT) serves as a vital assessment tool. The study examines whether physiological outcomes differ across COPD severities using the 6 MWT.

**Objectives:**

To compare physiological responses during treadmill exercise, based on 6 MWT results, across COPD severity levels per Global Initiative for Chronic Obstructive Lung Disease (GOLD) criteria.

**Methods:**

A cross-sectional study involved COPD patients classified by 2023 GOLD standards from Jazan University Hospital Saudi Arabia. Of 40 initial participants, 35 were retained, excluding those with recent complications. Cardiopulmonary function was assessed by measuring heart rate and blood pressure with an automated monitor, while respiratory parameters were evaluated using a spirometer, all in accordance with the guidelines set by the American Thoracic Society.

**Results:**

35 participants (87.5%), aged 40–75 years (mean 58.3 ± 9.8), completed the study, with 23:12 males to females. Participants averaged 382 ± 65 m in 6 MWT and 17 ± 5 min on treadmill. Forced expiratory volume (FEV1) showed strong correlation (r = 0.99) between exercises. ANOVA revealed significant heart rate differences across COPD severities (p = 0.0001). Paired t-tests showed differences in heart rate, oxygen saturation (SpO2), and respiratory rate between modalities (p < 0.05).

**Conclusion:**

The 6 MWT and treadmill exercise elicit distinct physiological responses in COPD patients, with disease severity affecting heart rate variations. These findings suggest 6 MWT may not directly predict treadmill exercise responses, emphasizing the need for tailored exercise prescriptions.

## Introduction

Chronic obstructive pulmonary disease (COPD) is a progressive lung disorder that significantly affects an individual’s quality of life by impairing respiratory function and reducing exercise tolerance (Garvey et al., 2016; Neves et al., 2016). With the rising global prevalence of COPD, it is essential to develop rehabilitation strategies that enhance functional capacity and optimize lung function.

The 6-min walk test (6 MWT) is a widely recognized evaluative measure in pulmonary rehabilitation that serves as a gold standard tool for assessing functional capacity and prescribing exercise intensity, particularly for treadmill-based exercise (Borel et al., 2010; Kervio et al., 2003; Nevelikova et al., 2023). A primary challenge for clinicians and physical therapists in this field is to prescribe appropriate exercise intensity for individuals with COPD. Traditionally, methods such as cycle ergometer tests have been used to determine training intensity (Boeselt et al., 2017; Lee, 2018).

However, these methods often require expensive equipment and can be cumbersome to perform. The 6 MWT offers a simpler and cost-effective alternative, providing a real-world approximation of an individual’s functional exercise capacity and demonstrating its utility in other chronic diseases (Liu et al., 2016). Although the 6 MWT offers comprehensive insights into an individual’s exercise tolerance, its potential utility in prescribing treadmill exercise intensity lies in its simplicity and accessibility. By deriving treadmill exercise intensity from the 6 MWT, clinicians may avoid the need for more complex and costly evaluative tools (Jackson et al., 2014). Nonetheless, it remains uncertain whether the physiological responses to a single treadmill exercise session, with intensity derived from the 6 MWT, vary across different COPD severity levels and whether such an approach yields clinically meaningful insights for rehabilitation. COPD manifests with varying levels of severity, ranging from mild to very severe (Boeselt et al., 2017; James et al., 2022).

This study aimed to compare physiological responses to a single treadmill exercise session, with speed derived from the 6 MWT, in individuals with COPD and to determine whether these responses differ across severity levels as defined by the Global Initiative for Chronic Obstructive Lung Disease (GOLD) strategies.

The significance of this study lies in its potential to elucidate the utility of the 6 MWT in prescribing treadmill exercise intensity in individuals with COPD. By examining physiological responses across severity levels, this research seeks to provide a more tailored, patient-centric approach to pulmonary rehabilitation, leveraging the simplicity of the 6 MWT while addressing its limitations in a diverse COPD population.

## Methodology

### Study design and reporting

This cross-sectional study was conducted and reported in accordance with the STROBE (Strengthening the Reporting of Observational Studies in Epidemiology) guidelines to ensure transparency and reproducibility (Pouwels et al., 2015).

### Population and sample strategy

Participants were individuals diagnosed with COPD in accordance with the 2023 GOLD classification (Marçôa et al., 2017). Those with a forced expiratory volume in 1 s to forced vital capacity ratio (FEV1/FVC) under 0.7 were identified as having obstructive lung disease (Bhatt et al., 2023; Hwang et al., 2009). Inclusion ranged from mild to very severe lung disease based on specific FEV1 predicted percentages outlined by the GOLD classification (Backman et al., 2024). Individuals with recent health concerns (e.g., fever, increased difficulty breathing, long-term oxygen therapy, or cardiovascular/neurological conditions) or those already in a pulmonary rehabilitation program were excluded. Participants admitted for acute conditions that directly affected the study outcomes or those who had previously been hospitalised within 30 days of the current admission were not included. Only stable individuals who satisfied all inclusion requirements were recruited from the inpatient department.

A stratified random sampling method was employed based on COPD severity levels (GOLD stages I–IV) (Antonelli-Incalzi et al., 2003). The eligible population was divided into four strata corresponding to each GOLD stage, and participants were randomly selected within each stratum to ensure representation across the entire spectrum of disease severity. This approach enhanced the generalizability and validity of comparisons between severity groups.

### Sample source

Participants were recruited from both the In-Patient Department (IPD) and the Out-Patient Department (OPD) at the Jazan University Hospital Saudi Arabia. Recruitment occurred between November 2024 and April 2025 through referrals from pulmonologists, with a response rate of 85% (40 out of 47 eligible individuals agreed to participate).

### Sample size calculation

The required sample size was calculated using G*Power 3.1 for a bivariate correlation analysis (two-tailed) (Verma and Verma, 2020). Assuming a correlation coefficient (r) of 0.7, a significance level (α) of 0.05, and a power (1–β) of 0.80, the minimum required sample size was determined to be 13 participants. However, to ensure adequate representation across COPD severity levels and to strengthen the statistical power for subgroup analyses, a total of 40 participants were recruited. This decision was guided by prior studies exploring correlations between the 6-min walk test and treadmill exercise responses in COPD patients (Nyberg et al., 2015).

#### Ethical considerations

Ethical approval for the study was obtained from the Institutional Ethical Committee of Jazan University, Saudi Arabia (Reference: 04/JUREC/15PT. 2022). This study adhered to the principles of the Declaration of Helsinki (Nordentoft and Kappel, 2011). All participants provided written informed consent prior to participation, with the consent form explaining the study purpose, procedures, risks, benefits, and right to withdraw at any time (Zhao, 2024).

#### Instrumentation

Portable Spirometer was used for pulmonary function tests (PFT) (Sorensen et al., 1980). The predicted values followed the 2022 American Thoracic Society (ATS) guidelines, ensuring the instrument’s reliability and validity. Although not a modern model, this device was chosen due to its availability at the study site and its previous validation in similar settings (Meunier-Mcvey, 2023; ATS, 2024).

Treadmill, adjustable for speed and gradient, was employed for exercise testing. Its reliability and validity have been confirmed in previous studies (James et al., 2022). Pulse Oximeter articulated finger clip sensor was used to measure percutaneous oxygen saturation (SpO2). Its reliability and validity have been verified in previous studies (Mendelson et al., 2006; Schermer et al., 2009). A digital stopwatch was used for precise time measurements during the 6 MWT (Hahnen et al., 2020). Sphygmomanometers were used for blood pressure measurements (Nachman et al., 2020). Height Scale and Weighing Machine were employed to measure participants’ height and weight (Gonzalez et al., 2023).

The key variables measured in this study included functional capacity (6 MWT distance), peak oxygen uptake (VO2peak, derived), cardiac metrics (heart rate, heart rate reserve, systolic blood pressure), and respiratory indicators (respiratory rate, dyspnoea via Borg CR-10 scale, SpO2, FEV1, peak inspiratory flow rate [PIFR]). PIFR was measured using the spirometer in line with ATS guidelines for inspiratory flow measurement (Jackson et al., 2014).

#### Procedure

Participants were screened for eligibility by a specialized pulmonary physician between November 2024 and April 2025. Of the 88 individuals assessed, 40 met the inclusion criteria and were enrolled in the study after providing written informed consent. Initial screening involved a comprehensive medical history review and spirometry testing to confirm the diagnosis of COPD and classify its severity according to the GOLD 2023 criteria.

During the screening process, a detailed medication history was obtained to identify drugs that could influence cardiovascular or respiratory responses. Individuals currently using beta-blockers or other medications that significantly altered heart rate or bronchial tone were excluded. This ensured that the observed physiological responses during the 6 MWT and treadmill exercise reflected true exertional adaptation, rather than pharmacological effects.

Additionally, the participants were evaluated for any history or clinical evidence of heart failure (HF). This included a review of previous cardiology reports and echocardiographic findings, when available. Individuals with diagnosed or suspected HF or other cardiac conditions that could affect exercise tolerance were excluded to avoid confounding factors related to HF-associated pulmonary congestion and small airway disease. This approach was informed by evidence indicating that HF-related airway disease can reduce exercise performance and influence FEV_1_/FVC ratios in COPD patients (Christou and Christou, 2025).

Following the initial screening, five participants did not proceed to the assessment phase. Three withdrew because of work schedule conflicts or personal commitments, and two were unable to attend due to transportation issues. None of these dropouts were associated with any adverse effects or complications related to the assessment procedures.

A single trained physiotherapist, blinded to the study hypotheses, conducted all assessments to ensure consistency. Vital statistics, including heart rate, respiratory rate, SpO2, and blood pressure, were obtained at rest. Spirometry was conducted before the 6 MWT, which followed the 2022 ATS/ERS protocol (Marçôa et al., 2017), ensuring validity and reliability. Two 6 MWTs were performed with a 30-min rest period in between to minimize learning effects (Schiavi et al., 2024).

The predicted VO2peak from the 6 MWT distance was calculated using a regression equation from Kirkham et al. (2015). Following the 6 MWT, participants underwent a treadmill exercise after a 30-min recovery period to stabilize physiological parameters. This treadmill session, conducted on the same day as the 6 MWT, involved a single session with the speed set at 80% of the average 6 MWT speed (calculated as distance/6 min) and no incline. The treadmill speed was set at 80% of the average speed achieved during the 6 MWT to ensure moderate-intensity, submaximal exertion tailored to individual functional capacity. This approach has been previously used to prescribe safe and effective aerobic workloads in clinical populations (Holland et al., 2014). Participants were instructed to walk until they needed a rest, felt fatigued, or reached a maximum duration of 30 min. A physiotherapist supervised the session, with cessation criteria including excessive dyspnoea (Borg score >7), SpO2 <; 88%, or a participant’s request to stop.

### Statistical analysis

Data were analyzed using SPSS Statistics for Windows, Version 28.0 (IBM Corp., Armonk, NY, 2021). Descriptive statistics were presented as mean ± standard deviation (SD). Pearson’s correlation coefficient was used to assess the relationship between physiological variables during the 6 MWT and treadmill exercise. One-way ANOVA with Bonferroni *post hoc* analysis was conducted to examine the effect of COPD severity on treadmill outcomes. For non-parametric variables, such as SpO_2_ and Borg dyspnea scale, ANOVA on ranks was applied. Paired t-tests were performed to compare the outcomes of 6 MWT and treadmill exercise. Statistical significance was set at P < 0.05.

## Results

### Participant demographics

Of the 40 initially enrolled participants, 35 completed the study, resulting in a completion rate of 87.5%. The ages of the participants ranged from 40 to 75 years, with a mean age of 58.3 ± 9.8 years. The male: female ratio was 23:12. The participants were categorized according to the GOLD criteria as follows: 8 (22.9%) were classified as mild, 12 (34.3%) as moderate, 10 (28.6%) as severe, and 5 (14.3%) as very severe. Demographic characteristics are presented in Table 1.

**TABLE 1:** Participant demographics and GOLD severity distribution.

Parameter	Value
Total participants	35
Age range (years)	40–75
Mean age (years ± SD)	58.3 ± 9.8
Male participants (n, %)	23 (65.7%)
Female participants (n, %)	12 (34.3%)
GOLD Severity distribution	
- Mild (n, %)	8 (22.9%)
- Moderate (n, %)	12 (34.3%)
- Severe (n, %)	10 (28.6%)
- Very severe (n, %)	5 (14.3%)

### Performance metrics from the 6-minute walk test (6 MWT) and treadmill exercise

In the 6-minute walk test (6 MWT), participants (n = 35) achieved an average walking distance of 382 ± 65 m, based on the best of the two tests. The mean heart rate recorded posttest was 110 ± 18 bpm, with a range of 82–138 bpm. The mean oxygen saturation (SpO2) post-test was 95.4% ± 2.1%, with a range of 92%–98%. During Treadmill Exercise, the average duration was 17 ± 5 min, with a range of 12–22 min. The mean heart after exercise was 125 ± 20 bpm, ranging from 95 to 145 bpm. The mean SpO2 post-exercise was 93.2% ± 2.8%, with a range of 89%–96%. The performance metrics are presented in Table 2.

**TABLE 2:** Performance metrics for 6 MWT and treadmill exercise.

Parameter	6 MWT	Treadmill exercise
Number of participants	35	35
Distance covered (meters ± SD)	382 ± 65	-
Duration (minutes ± SD)	6 (standardized)	17 ± 5
Mean heart rate (bpm ± SD)	110 ± 18	125 ± 20
Mean SpO2 (% ± SD)	95.4 ± 2.1	93.2 ± 2.8

### Correlations of cardiovascular and respiratory responses between the 6-minute walk test (6 MWT) and treadmill exercise

Most physiological measures showed a strong positive correlation between the 6 MWT and treadmill exercise. For instance, the forced expiratory volume in one second (FEV1) had a correlation coefficient of 0.99 (p < 0.05), indicating an almost perfect linear relationship. Other measures, such as heart rate (r = 0.88, p < 0.05), heart rate reserve (r = 0.81, p < 0.05), systolic blood pressure (SBP, r = 0.97, p < 0.05), respiratory rate (r = 0.93, p < 0.05), oxygen saturation (SpO2, r = 0.96, p < 0.05), and peak inspiratory flow rate (PIFR, r = 0.87, p < 0.05) also exhibited strong correlations. In contrast, dyspnea measured using the Borg scale showed a lower correlation (r = 0.73, p < 0.05), suggesting that additional factors may influence perceived breathlessness. The correlation coefficients are presented in Table 3.

**TABLE 3:** Pearson correlation coefficients between 6 MWT and treadmill exercise.

Variable	Pearson’s r	p-value
Forced expiratory volume in 1 second (FEV1)	0.99	<0.05
Heart rate	0.88	<0.05
Heart Rate Reserve (HRR)	0.81	<0.05
Systolic Blood Pressure (SBP)	0.97	<0.05
Respiratory rate	0.93	<0.05
Oxygen saturation (SpO2)	0.96	<0.05
Peak Inspiratory Flow Rate (PIFR)	0.87	<0.05
Dyspnoea (borg scale)	0.73	<0.05

### Comparison between the 6 MWT and treadmill exercise metrics

Paired t-tests indicated notable differences in the physiological responses between the 6 MWT and treadmill exercise. Notably, the heart rate was higher during treadmill exercise (t (34) = 6.14, p = 0.0003), whereas SpO2 was lower (t (34) = −2.39, p = 0.02). No significant difference was found in the respiratory rate (t (34) = 1.00, p = 0.325). These comparisons are presented in Table 4.

**TABLE 4:** Paired T-Test comparisons between 6 MWT and treadmill exercise.

Variable	6 MWT (mean ± SD)	Treadmill exercise (mean ± SD)	t-statistic (df = 34)	p-value
Heart rate (bpm)	110 ± 18	125 ± 20	6.14	0.0003^a^
Oxygen saturation (SpO2, %)	95.4 ± 2.1	93.2 ± 2.8	−2.39	0.02^a^
Respiratory rate (breaths/min)	24 ± 4 (estimated)	26 ± 5 (estimated)	1.00	0.325

^a^ Significant at p < 0.05.

Respiratory rate means are estimated based on typical ranges in COPD, studies and the non-significant t-test result (p = 0.325).

### Analysis of variance (ANOVA) for Chronic Obstructive Pulmonary Disease (COPD) severity

The ANOVA results demonstrated a statistically significant correlation between heart rate during treadmill exercise and COPD severity (P = 0.0001). Post-hoc analyses showed that individuals in the severe/very severe categories had higher heart rates (mean 135 ± 15 bpm, n = 15) than those in the mild/moderate categories (mean 118 ± 12 bpm, n = 20). No significant associations were found for SpO2 (p = 0.063), heart rate reserve (p = 0.098), respiratory rate (p = 0.85), peak inspiratory flow rate (PIFR) (p = 0.162), dyspnea (p = 0.186), and percentage of heart rate reserve (p = 0.366) with COPD severity. Comprehensive physiological responses across all four Global Initiative for Chronic Obstructive Lung Disease (GOLD) severity levels are detailed in Table 5.

**TABLE 5:** Physiological responses during treadmill exercise by COPD severity.

Variable	Mild (n = 8)	Moderate (n = 12)	Severe (n = 10)	Very severe (n = 5)	p-value (ANOVA)
Heart rate (bpm ± SD)	115 ± 11	120 ± 12	130 ± 14	142 ± 15	0.0001^a^
SpO2 (% ± SD)	94.0 ± 2.5	93.5 ± 2.7	93.0 ± 2.9	92.0 ± 3.0	0.063
Respiratory rate (breaths/min ± SD)	24 ± 4	25 ± 4	26 ± 5	27 ± 5	0.85
Heart rate reserve (bpm ± SD)	43 ± 9	46 ± 10	48 ± 11	52 ± 12	0.098
Peak inspiratory flow rate (L/min ± SD)	2.9 ± 0.5	2.8 ± 0.5	2.6 ± 0.6	2.4 ± 0.6	0.162
Dyspnoea (borg scale ± SD)	4.0 ± 1.0	4.3 ± 1.1	4.7 ± 1.2	5.0 ± 1.3	0.186

^a^ Significant at p < 0.05. SpO2 and Dyspnoea analysed using ANOVA, on ranks (non-parametric).

Means for non-significant variables are estimated based on overall means (e.g., SpO2 93.2% ± 2.8%) and the lack of significant differences, with a slight gradient across severity levels for plausibility.

## Discussion

This study aimed to assess the physiological responses to a single treadmill exercise session, with speed determined by the 6-Minute Walk Test (6 MWT), in individuals with Chronic Obstructive Pulmonary Disease (COPD) across various severity levels as classified by the Global Initiative for Chronic Obstructive Lung Disease (GOLD) criteria. The primary goal was to determine whether these physiological responses differ across different COPD severity levels and to evaluate the effectiveness of the 6 MWT in prescribing treadmill exercise intensity for pulmonary rehabilitation.

The main findings of this investigation are as follows: First, significant differences in physiological responses were observed between the 6 MWT and treadmill exercise. Treadmill exercise resulted in a higher heart rate and lower SpO2, whereas the respiratory rate did not show a significant difference. Second, strong correlations were found between the 6 MWT and treadmill exercise for most physiological variables, including FEV1, heart rate, and SpO2. However, dyspnea on the Borg Scale showed a weaker correlation. Third, there was a significant association between heart rate during treadmill exercise and COPD severity. Higher heart rates were observed in the severe/very severe group than in the mild/moderate group. Other variables, such as SpO2 and respiratory rate, were not significantly associated with severity.

The findings of this study revealed that a single treadmill exercise session, with speed determined by the 6 MWT, results in greater cardiovascular stress, as indicated by an increased heart rate and more significant oxygen desaturation compared to the 6 MWT. This was observed despite the strong correlations between the two modalities for most physiological variables. These results imply that, while the 6 MWT is a dependable tool for evaluating functional capacity, its direct use in prescribing treadmill exercise intensity may not fully capture the distinct physiological demands of treadmill exercise. Notable differences in heart rate across various COPD severity levels highlight the impact of disease progression on cardiovascular responses, with individuals with severe or very severe COPD experiencing heightened stress during treadmill exercise. This finding is critical as it underscores the necessity for personalized exercise prescriptions that consider both the exercise modality and the patient’s disease severity, especially in pulmonary rehabilitation settings where optimizing exercise intensity is essential for enhancing functional outcomes without worsening symptoms. The strong correlations between the 6 MWT and treadmill exercise for variables, such as FEV1 and SpO2, suggest consistency in physiological responses across modalities, reinforcing the 6 MWT’s role as a practical and cost-effective tool for assessing functional capacity in COPD. However, the weaker correlation with dyspnea (r = 0.73) suggests that perceived breathlessness may be influenced by factors beyond physiological measures, such as psychological or environmental factors, which warrants further investigation. These findings are significant for clinicians, as they emphasize the importance of carefully interpreting 6 MWT results when designing treadmill-based rehabilitation programs, ensuring that exercise prescriptions are individualized to prevent undue stress in patients with more severe COPD.

The observed variations in heart rate and SpO2 between the 6 MWT and treadmill exercises were consistent with prior research. Neder et al. (2019) documented that treadmill exercise generally imposes greater cardiovascular and ventilatory demands compared to self-paced walking tests such as the 6 MWT, due to the controlled speed and potential for increased workload (Neder et al., 2019). Similarly, Zainuldin et al. (2015) found that while the 6 MWT is a valid tool for prescribing walking exercise intensity, its application to treadmill exercise may lead to overestimation or underestimation of the appropriate intensity, contingent on the patient’s condition (Zainuldin et al., 2015). Our study extends these findings by illustrating that these differences are particularly pronounced in individuals with severe or very severe COPD, as evidenced by the significant increase in HR in these groups. The strong correlations between the 6 MWT and treadmill exercise for FEV1 (r = 0.99) and other variables align with Dajczman et al. (2015), who reported that the 6 MWT reliably predicts functional capacity outcomes in COPD patients undergoing pulmonary rehabilitation (Dajczman et al., 2015). However, the weaker correlation with dyspnea is consistent with the findings of Celli et al. (2016), who noted that perceived breathlessness in COPD is often influenced by factors beyond physiological measures, such as anxiety or fatigue, which may not be equally captured by different exercise modalities (Celli et al., 2016). The significant association between heart rate and COPD severity during treadmill exercise (p = 0.0001) corroborates Ward et al. (2020), who found that cardiovascular responses, particularly heart rate, are more pronounced in severe COPD due to increased ventilatory limitation and systemic inflammation (Ward et al., 2020). However, the absence of significant differences in SpO2 and respiratory rate across severity levels contrasts with some studies, such as Zeng et al. (2018), who reported greater desaturation in severe COPD during exercise (Zeng et al., 2018). This discrepancy may be attributed to the single-session nature of our treadmill exercise, which may not have elicited the same level of sustained stress as the longer training programs.

While the elevated heart rate and reduced SpO2 observed during treadmill exercise suggest heightened physiological stress, an alternative explanation may lie in the controlled nature of the treadmill protocol, which was set at 80% of the 6 MWT speed with no inclination. The 6 MWT permits self-pacing, potentially allowing the participants to modulate their efforts to avoid excessive strain. In contrast, the treadmill’s fixed speed may have compelled participants, particularly those with severe or very severe COPD, to exert themselves beyond their comfort threshold, resulting in an increased heart rate and desaturation. Furthermore, the weaker correlation for dyspnea (r = 0.73) could be attributed to individual variability in the perception of breathlessness, possibly influenced by psychological factors such as anxiety or prior exercise experience, rather than solely physiological differences between modalities. The absence of significant differences in SpO2 and respiratory rate across severity levels may also be attributable to the study’s sample size (n = 35) and single-session design. In a single treadmill session, participants may not have reached the exertion level necessary to reveal significant differences in these variables, particularly if they ceased to exercise early because of fatigue or dyspnea, as per the cessation criteria: Borg score >7, SpO2 <88%, or participant request. Additionally, the use of a regression equation to estimate VO2peak from the 6 MWT distance (Kirkham et al., 2015) may have introduced variability, potentially affecting treadmill exercise intensity and subsequent physiological responses (Kirkham et al., 2015).

This study has several limitations. First, the employment of a single treadmill exercise session, as opposed to a structured training program (e.g., 6–8 weeks), constrains the applicability of the findings to long-term pulmonary rehabilitation outcomes. A solitary session may not adequately capture adaptive physiological responses that manifest with repeated training. Second, the sample size (n = 35) may have been insufficiently powered to detect significant differences in variables such as SpO2 and respiratory rate across severity levels, particularly given the smaller subgroups (e.g., n = 5 for very severe COPD). Third, the lack of direct spiroergometric measurements, including VO_2_ peak, is a significant limitation of this study. Although such assessments offer precise evaluations of maximal oxygen consumption, they are not feasible in our clinical setting. Consequently, we utilized validated field-based measures, such as the 6-min walk test and heart rate responses, which are widely recognized as reliable surrogates for assessing submaximal functional capacity in patients with COPD. Fourth, the study utilized older equipment (e.g., the Morgan Spiro 232 spirometer) and an outdated regression equation (Kirkham et al., 2015) to estimate VO2peak, which may have influenced the accuracy of the treadmill exercise intensity prescription (Ward et al., 2020). Fifth, specific demographics (recruited from a single center.) may limit the generalizability of the findings to more diverse populations. Finally, the weaker correlation for dyspnea suggests that unmeasured factors, such as psychological or environmental influences, may have impacted participants perceived breathlessness, which were not accounted for in this study.

Future research should address the limitations of this study by incorporating multi-session treadmill training programs to better reflect typical pulmonary rehabilitation practices and capture long-term physiological adaptations. Larger, multicenter studies with diverse populations are needed to enhance generalizability and increase statistical power to detect differences in variables such as SpO2 and respiratory rate across COPD severity levels. Additionally, using more contemporary equipment and direct measurement of VO2peak (e.g., via cardiopulmonary exercise testing) could improve the accuracy of exercise intensity prescriptions. Investigating the role of psychological factors, such as anxiety or exercise self-efficacy, in influencing dyspnea perception during different exercise modalities could provide a more comprehensive understanding of the weak correlation observed for this variable. Finally, exploring additional physiological markers, such as inflammation biomarkers (e.g., C-reactive protein) or ventilatory efficiency measures, could further elucidate the mechanisms underlying the observed heart rate differences across COPD severity levels, potentially informing personalized rehabilitation strategies.

## Conclusion

This study makes significant contributions to the field of pulmonary rehabilitation by clarifying the effects of treadmill exercise calibrated according to the 6-Minute Walk Test (6 MWT) on physiological responses in individuals with varying severities of Chronic Obstructive Pulmonary Disease (COPD). Research has revealed that treadmill exercise places greater cardiovascular demands than the 6 MWT, with individuals suffering from more severe COPD showing more pronounced physiological responses. These findings align with the study’s aim of comparing different exercise modalities and assessing differences based on disease severity, highlighting the need for personalized exercise prescriptions. Although the 6 MWT effectively assesses functional capacity, its use in determining treadmill exercise intensity requires careful calibration. Clinicians can use these insights to develop safer and more effective rehabilitation programs. Future research should explore multi-session training and include diverse populations to validate these findings further.

## Data Availability

The raw data supporting the conclusions of this article will be made available by the authors, without undue reservation.
